# Modelling the Shear Banding in Gradient Nano-Grained Metals

**DOI:** 10.3390/nano11102468

**Published:** 2021-09-22

**Authors:** Tianyu Chen, Jianjun Li

**Affiliations:** 1 College of Mechanical and Electrical Engineering, Central South University, Changsha 410083, China; cty9525785@mail.nwpu.edu.cn; 2 State Key Laboratory of High Performance Complex Manufacturing, Central South University, Changsha 410083, China

**Keywords:** gradient nano-grained metal, shear band, ductility, strain delocalization

## Abstract

Extensive experiments have shown that gradient nano-grained metals have outstanding synergy of strength and ductility. However, the deformation mechanisms of gradient metals are still not fully understood due to their complicated gradient microstructure. One of the difficulties is the accurate description of the deformation of the nanocrystalline surface layer of the gradient metals. Recent experiments with a closer inspection into the surface morphology of the gradient metals reported that shear bands (strain localization) occur at the surface of the materials even under a very small, applied strain, which is in contrast to previously suggested uniform deformation. Here, a dislocation density-based computational model is developed to investigate the shear band evolution in gradient Cu to overcome the above difficulty and to clarify the above debate. The Voronoi polygon is used to establish the irregular grain structure, which has a gradual increase in grain size from the material surface to the interior. It was found that the shear band occurs at a small applied strain in the surface region of the gradient structure, and multiple shear bands are gradually formed with increasing applied load. The early appearance of shear banding and the formation of abundant shear bands resulted from the constraint of the coarse-grained interior. The number of shear bands and the uniform elongation of the gradient material were positively related, both of which increased with decreasing grain size distribution index and gradient layer thickness or increasing surface grain size. The findings are in good agreement with recent experimental observations in terms of stress-strain responses and shear band evolution. We conclude that the enhanced ductility of gradient metals originated from the gradient deformation-induced stable shear band evolution during tension.

## 1. Introduction

The strength-ductility trade-off is a longstanding problem with metals, which means that the achievement of high strength in the materials is usually accompanied by low ductility, and vice versa [1,2,3,4]. In the last decade, inspired by biological materials such as bamboo and shell [5], extensive experiments have demonstrated that this dilemma can be solved by introducing gradient nanostructures into the materials [6,7,8,9,10,11]. A typical example is a gradient nano-grained metal, which is characterized by a gradual change in grain size from tens of nanometers in the material surface to tens of micrometers in the center. In the pioneering work of Fang et al. [6], gradient nano-grained Cu produced by a surface mechanical grinding treatment achieved a yield strength of 129 MPa, which doubled the yield strength of the coarse-grained (CG) Cu. Meanwhile, the gradient Cu preserved a ductility comparable to that of the CG Cu. This excellent strength–ductility synergy of gradient nano-grained metals is believed to be attributed to unique gradient nanostructures, which not only combines the high strength of the nanograins and the high ductility of the coarse grains, but also confers on the gradient metals extra strain hardening through hetero-deformation induced strengthening [12,13]. Moreover, the gradient structure can also provide metals, including stainless steels [14,15,16] and alloys [17], with other superior mechanical properties, such as enhanced fatigue resistance [15,16,18] and lower coefficients of friction [17,19]. 

Although the deformation mechanisms of the gradient metals have been studied extensively [20,21,22,23,24], it is still not fully understood how the nano-grained (NG) layer at the surface of the gradient materials deforms during tension, especially considering that homogeneous NG metals usually have very limited ductility [25,26] and often fail through the formation of shear bands [27,28,29] that are initiated at a very small plastic strain, e.g., 0.3% [30], similar to the failure mechanism of brittle metallic glasses [31,32,33]. Recently, experiments showed that the NG layer in the gradient metals does not deform uniformly under tension as suggested in the previous studies [6,34]. Instead, strain localizations, such as shear bands, are formed at the surface of the gradient metals [35,36,37,38]. For example, Yuan et al. [35] found that two shear bands were formed at the surface NG region of gradient nanostructured interstitial-free (IF) steel under a very small applied strain, i.e., 1%. However, rather than quickly evolving across the whole material and inducing fracture, the shear bands in the gradient IF steel became stabilized during tension. The gradient IF steel finally attained a uniform elongation of 20.6%, comparable to its coarse-grained (CG) counterpart [35]. Similarly, Wang et al. [37] observed dense dispersed shear bands on the surface of gradient structured Ni under tension, while the gradient Ni still maintained a high uniform elongation of 30.6% [37]. 

In the present study, in order to further study shear band evolution and how the shear band patterns are affected by microstructures in the gradient metals, we built a computational model to simulate the shear band formation process in gradient nano-grained Cu. A dislocation density-based constitutive law was employed to model the gradient Cu, in which strain softening was introduced in the surface NG region. Voronoi tessellation was used to generate the grain size gradient microstructure of the material. Our simulation results show that, contrary to the few shear bands formed in the homogeneous NG Cu, up to 50 shear bands were formed in the gradient Cu. A detailed analysis of the shear band evolution proved that the shear bands were stabilized in the gradient metal. Moreover, the effect of microstructures of the gradient metals on the shear band morphologies was studied. The number of shear bands decreased with the increase of grain size distribution index, increase of grain size gradient region thickness, and decrease of the surface grain size. Our simulation results are in good agreement with experimental data in terms of both the stress-strain curve and shear band patterns. 

## 2. Model Description 

### 2.1. Constitutive Model for Gradient Cu

**Figure 1:**
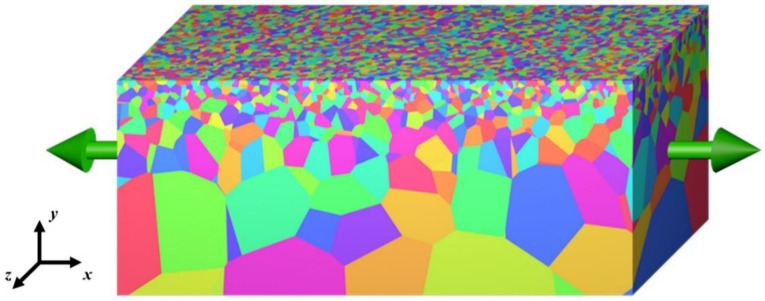
Schematic of a computational model for gradient nano-grained Cu.

Figure 1 shows a schematic of a computational model of the gradient nano-grained Cu. The dislocation-based constitutive relation developed by Li et al. [39,40,41,42] was employed to model the gradient metal. In the model, the gradient metal is treated as a composite composed of a coarse-grained (CG) core and a grain size gradient surface layer (GSL), inside which the grain size variation occurs. The GSL is further divided into multiple homogeneous layers, each with a uniform grain size distribution but different grain sizes [39,40,41]. The essential constitutive equations in the rate form for each layer, including the CG core, are summarized as follows. The total strain rate ${\dot{\varepsilon }}_{ij}$ can be expressed as the superposition of the elastic strain rate ${\dot{\varepsilon }}_{ij}^{e}$ and the plastic strain rate ${\dot{\varepsilon }}_{ij}^{p}$ as:

(1) $${\dot{\varepsilon }}_{ij}={\dot{\varepsilon }}_{ij}^{e}+{\dot{\varepsilon }}_{ij}^{p},$$

where the elastic strain rate ${\dot{\varepsilon }}_{ij}^{e}$ follows Hooke’s Law as:

(2) $${\dot{\varepsilon }}_{ij}^{e}=\frac{1+\nu }{E}\left({\dot{\sigma }}_{ij}-\frac{\nu }{1+\nu }{\dot{\sigma }}_{kk}\delta_{ij}\right),$$

where *E*, *ν*, ${\dot{\sigma }}_{ij}$ and $\delta_{ij}$ are the elastic modulus, Poisson’s ratio, stress rate, and Kronecker’s delta, respectively. The plastic part of the strain rate ${\dot{\varepsilon }}_{ij}^{p}$ is assumed to obey the *J*_2_ flow theory:

(3) $${\dot{\varepsilon }}_{ij}^{p}=\eta \sigma_{ij}^{\prime },$$

where $\sigma_{ij}^{\prime }=\sigma_{ij}-\sigma_{kk}/3$ is the deviatoric stress. $\eta =3{\dot{\varepsilon }}^{p}/2\sigma$ is a material parameter that can be extracted from uniaxial tensile test. Here ${\dot{\varepsilon }}^{p}=\sqrt{2{\dot{\varepsilon }}_{ij}^{p}:{\dot{\varepsilon }}_{ij}^{p}/3}$ and $\sigma =\sqrt{3{{\dot{\sigma }}^{\prime }}_{ij}:{{\dot{\sigma }}^{\prime }}_{ij}/2}$ are the von Mises equivalent plastic strain rate and the von Mises equivalent stress, respectively. By employing the modified KME model [39], the von Mises equivalent stress *σ* in each homogeneous layer can be expressed as:

(4) $$\sigma =\sigma_{0}+M\alpha \mu b\sqrt{\rho_{s}}+k_{\mathrm{HP}}/d,$$

where $\sigma_{0}$, *M*, *α*, *μ*, *b*, $\rho_{s}$, $k_{\mathrm{HP}}$, and *d* are the lattice friction stress, Taylor factor, Taylor constant, magnitude of Burgers vector, shear modulus, statistically stored dislocation density, Hall-Petch constant, and grain size, respectively. Compared with the original KME model [43,44], two additional terms, i.e., the first and the third terms on the right-hand side of Equation (4), are introduced in this modified version. The first term $\sigma_{0}$ accounts for the lattice friction. The last term $k_{\mathrm{HP}}/d$, i.e., Hall-Petch strengthening, is introduced due to the presence of the grain boundaries that serve as obstacles for dislocation movement [45]. Generally, when metals are deformed, dislocations pile up at the grain boundaries, generating stress concentration in the neighboring grains to active dislocation sources. In small grains, fewer dislocations pile up at the grain boundaries, leading to weaker stress concentration. Therefore, greater applied stress is required to further deform the material when the grain size is small, resulting in the grain size-dependent yield strength. The evolution of statistically stored dislocation with plastic strain $\varepsilon^{p}$ is assumed to follow:

(5) $$\frac{d\rho_{s}}{d\varepsilon^{p}}=M\left(k+k_{1}\sqrt{\rho_{s}}-k_{2}\rho_{s}-k_{e}\rho_{s}\right),$$

where $k=k_{3}/\left(bd\right)$, $k_{1}=\psi /b$, $k_{2}=k_{20}{\left({{\dot{\varepsilon }}^{p}/\dot{\varepsilon }}_{0}\right)}^{-1/n_{0}}$ and $k_{e}={\left(d_{e}/d\right)}^{2}$. Here $k_{3}$ is a geometric factor, *ψ* is a proportionality factor, $k_{20}$ and ${\dot{\varepsilon }}_{0}$ are material constants, $n_{0}$ is inversely proportional to temperature, and $d_{e}$ is the reference grain size. The last term on the right-hand side of Equation (5) is an additional term, as compared with the original KME equation, to account for the extra dislocation dynamic recovery in nanograined or ultrafine-grained Cu. This term was first introduced by Li et al. [39].

According to Li et al.’s model [39,40,41], two sets of material parameter values are used, which separately describe the nanocrystalline region with grain sizes ranging from tens to hundreds of nanometers, and the microcrystalline region with grain sizes ranging from hundreds of nanometers to tens of micrometers. The values of the material parameters are summarized in Table 1. The references for determining these values are as follows. For the gradient nano-grained Cu, existing experimental measurements show that the Young’s modulus remains almost the same as the grain size changes [46]. Therefore, identical values of Young’s modulus were used for both the nanocrystalline and microcrystalline models. For FCC metals such as the copper used here, the value of the Taylor factor *M* was adopted as 3.06, according to Kocks [47]. The value for the dynamic recovery constant 2 $n_{0}$ was adopted from Kim et al.’s work on the modelling of fine-grained Cu [48]. The values for the Hall-Petch slope $k_{\mathrm{HP}}$ and the lattice frictional stress $\sigma_{0}$ of the microcrystalline Cu were adopted from experimental data [49]. A previous experimental study [26] showed that $k_{\mathrm{HP}}$ decreases and $\sigma_{0}$ increases as grain size decreases into the nanocrystalline region for copper. Thus, a smaller $k_{\mathrm{HP}}$ and a higher $\sigma_{0}$ were used for the nanocrystalline Cu. The values for the remaining parameters, where discrepancies existed between the nanocrystalline model and the microcrystalline model, were determined by fitting the experimental stress-strain curves of Cu with different grain sizes, i.e., 30 nm [50], 200 nm [2], 500 nm [51], and 36 μm [36]. These parameters include the Taylor factor *α*, proportionality factor *ψ*, dynamic recovery factor *k*_2_, dynamic recovery constant 1 *k*_20_, geometric factor *k*_3_, reference grain size *d*_e_, and initial dislocation density $\rho_{0}$. Note that according to the experimental data [52,53,54], in the gradient metals produced by surface severe plastic deformation, such as surface mechanical attrition treatment, the nanocrystallized surface region has a much higher dislocation density, usually two orders higher than that of the untreated coarse-grained matrix. Therefore, considering the huge difference in dislocation density between the nanocrystalline and microcrystalline regions, zero initial dislocation density was adopted for the microcrystalline Cu in our simulations.

**Figure 2:**
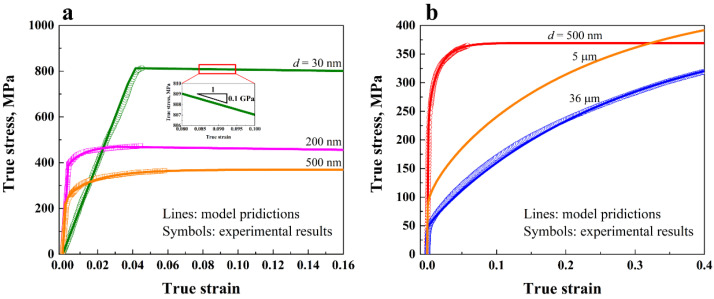
Comparison between model predictions and experimental results for true stress-strain curves of (**a**) NC Cu and (**b**) MC Cu. Inset in (**a**) shows the linear softening in the NC region.

Figure 2 compares the model predictions and experimental results for true stress-strain curves of the NC Cu and MC Cu. The results show that the model is capable of accurately reproducing the mechanical responses of Cu with various grain sizes. Note that in order to capture the deformation-induced strain softening in the surface of the gradient metal [34,36], linear softening was adopted for the NC Cu after the uniform elongation was reached, as illustrated in the inset of Figure 2. This simple form of linear softening was successfully employed to study the shear band formation in Al/SiC nanolayers [55]. Here, the softening slope was adopted as 0.1 GPa based on the experimental measurements [34,56,57], where the softening slope of the nanocrystalline Cu under tension or compression was between 0.1 GPa and 1 GPa.

### 2.2. Generation of the Gradient Microstructure

**Figure 3:**
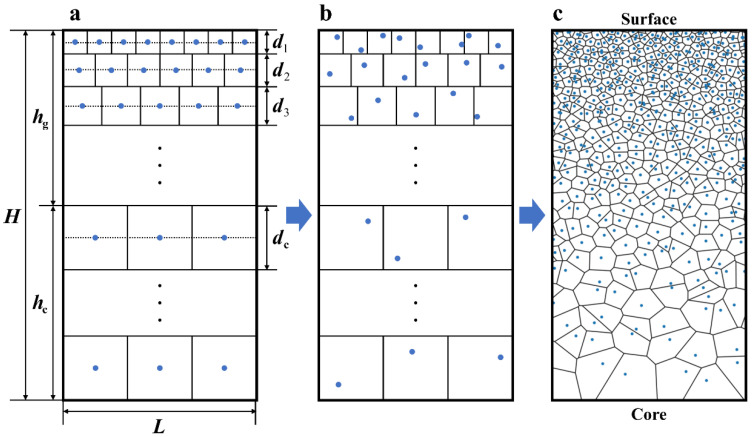
The generation process of the grain size gradient microstructure of the gradient Cu using the Voronoi tessellation: (**a**) initial seed positions, (**b**) perturbed seed positions, and (**c**) schematic of the generated gradient grains.

During deformation, shear bands preferentially form at sites where stress concentration occurs [37,58]. Usually, stress concentration appears at the interfaces between materials with contrasting mechanical properties, as shown in molecular dynamics simulations [59,60]. Hence in gradient nano-grained metals, the stress concentration locations pertain primarily to grain boundaries between grains with different grain sizes, which can also be observed from the stress distribution maps obtained from molecular dynamics simulations [61,62]. Therefore, in the current finite element model, the grain size gradient microstructure of the gradient nano-grained Cu was explicitly generated using the Voronoi tessellation method. Figure 3 summarizes the generation process, where the blue dots correspond to the seeds, namely the control points, of each grain. First, the seeds are put at the center of the ideally arranged grains (Figure 3a). Then, perturbations are applied to the initial seed positions (Figure 3b), so that random grain boundaries can be developed, as shown in Figure 3c. As indicated in Figure 3a, the grain size of the $i^{\mathrm{th}}$ layer and the CG core are denoted by $d_{i}$ and $d_{c}$, respectively. According to the experimental data [9,63], the grain size distribution along the thickness direction in the gradient Cu follows the relation:

(6) $$\mathrm{lg}\left(d\right)=a_{g}h^{n}+b_{g},$$

where *h* and *n* are the distance from the surface of the material and the grain size distribution index, respectively, and $a_{g}$ and $b_{g}$ are two constants. Once $d_{1}$, $d_{c}$, *n*, and the number of layers in the GSL (*k*) are specified, $a_{g}$ and $b_{g}$, as well as the grain sizes of the other layers ($d_{k}$) in the GSL, can be determined by solving the following equations, obtained by considering the geometrical constraints between layers with different grain sizes:

(7) $$\left\{ \begin{array}{l} \mathrm{lg}\left(d_{1}\right)=a_{g}{\left(d_{1}/2\right)}^{n}+b_{g}\\ \mathrm{lg}\left(d_{2}\right)=a_{g}{\left(d_{1}+d_{2}/2\right)}^{n}+b_{g}\\ \vdots \\ \mathrm{lg}\left(d_{k}\right)=a_{g}{\left(d_{1}+d_{2}+\cdots +d_{k}/2\right)}^{n}+b_{g}\\ \mathrm{lg}\left(d_{c}\right)=a_{g}{\left(d_{1}+d_{2}+\cdots +d_{k}+d_{c}/2\right)}^{n}+b_{g} \end{array}\right.,$$

Equation (7) can be solved numerically using MATLAB. Here the grain size of the *k*^th^ layer ($d_{k}$) is the value calculated from Equation (6) at the center of the *k*^th^ layer, i.e., at the positions marked by the dashed lines in Figure 3a.

### 2.3. The Finite Element Model for the Gradient Cu

**Figure 4:**
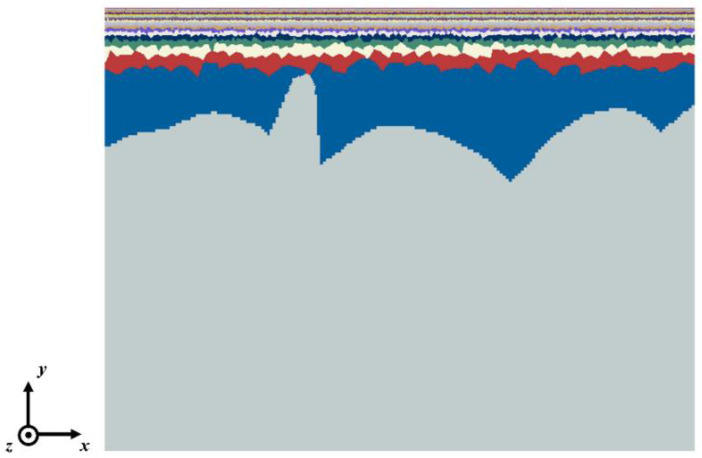
A typical finite element model with 28 homogeneous layers where grain boundaries between layers with different grain sizes are modeled.

Commercial finite element software, ABAQUS, was used here to conduct simulations. Both 2D and 3D models were established for calculations. Figure 4 shows a typical 2D finite element model with 28 homogeneous layers, where grain boundaries between layers with different grain sizes are modeled. For the sake of computational efficiency, the grain boundaries within each layer were eliminated. Our simulations show that this simplification did not affect the final results because it is the grain boundaries between the layers that induce strain localization and then the shear bands. In the 2D model, the length of the model *L* (in the *x*-direction; refer to Figure 1 for the coordinate system) was 200 μm and the total thickness of the model *H* (in the *y*-direction) was 150 μm. Symmetrical boundary conditions were used at the left and bottom boundaries, and a displacement boundary condition was applied at the right boundary. Four-node plane strain elements (CPE4; *n* = 568,916) and 1308 three-node plane strain elements (CPE3) were used in the 2D simulations. Detailed geometrical parameters for the 2D model are summarized in Table 2. For the 3D model, considering the computational limitation, the length *L* (in the *x*-direction), thickness *H* (in the *y*-direction), and width *W* (in the *z*-direction) of the model were reduced to 4 μm, 50 μm and 1 μm, respectively. Because of the limited dimensions of the 3D model, the periodic boundary condition was applied at the *x* and *z* directions. The symmetric boundary condition was applied at the bottom plane. Eight-node reduced integration linear brick elements (C3D8R; *n* = 376,050) were used in the 3D simulation. Due to the huge computation cost, the 3D simulation was only conducted for verification of the model. 

## 3. Results and Discussions

### 3.1. Shear Band Multiplication in Gradient Nano-Grained Cu

**Figure 5:**
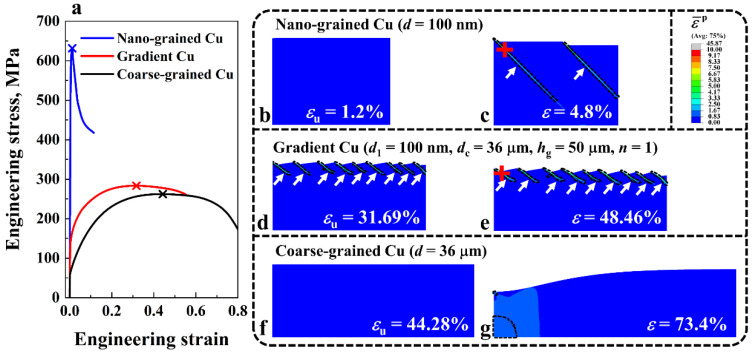
(**a**) Engineering stress-strain curves of the NG, gradient, and CG Cu. (**b**–**g**) Deformation behavior of the NG, gradient, and CG Cu during tension. Regions where the equivalent plastic strain ${\bar{\varepsilon }}^{p}$ is larger than 1 are enclosed by black dashed lines.

Figure 5a shows the engineering stress-strain curves of the NG, gradient and CG Cu. The points where the uniform elongation $\varepsilon_{u}$ is reached are denoted by the symbol ‘×’. The grain size of the NG and the CG Cu are 100 nm and 36 μm, respectively. For the gradient structure, the surface grain size *d*_1_, core grain size *d*_c_, GSL thickness *h*_g_, and the grain size distribution index *n* were adopted as 100 nm, 36 μm, 50 μm and 1, respectively. The deformation behaviors of the three structures are compared in Figure 5b–g. Regions with the equivalent plastic strain larger than 1 are enclosed by black dashed lines to highlight the strain localization zone. Note that in this section, in order to induce nonlinear deformation in the homogeneous structures, a notch with 0.5 μm in depth and 5 μm in width was introduced at the top left corner of the three structures. As illustrated in Figure 5a, the homogeneous NG and CG structures have the problem of strength-ductility trade-off. That is, the NG Cu has a high yield strength ($\sigma_{Y}=606.88\;\mathrm{MPa}$) but very limited ductility ($\varepsilon_{u}=1.2\%$), while the CG Cu has a low yield strength ($\sigma_{Y}=61.81\;\mathrm{MPa}$) but high ductility ($\varepsilon_{u}=44.28\%$). This conundrum was solved in the gradient structure, which possesses a high yield strength of 131.68 MPa and a uniform elongation ($\varepsilon_{u}=31.69\%$) comparable to that of the CG structure.

In order to explain the enhanced strength-ductility of the gradient structure, especially how the large plastic strain was accommodated in the NG layers of the gradient structure, the deformation behavior of the NG, gradient, and CG Cu were analyzed. As shown in Figure 5b–g, the three structures exhibited distinct deformation behaviors. For the NG structure, only two shear bands (indicated by the white arrows) were formed in the material. Even at a very small, applied strain, e.g., ε=4.8% (Figure 5c), the shear bands had fully formed and extended across nearly the whole structure, resulting in the low ductility of the NG Cu. This phenomenon is similar to those observed in molecular dynamics simulations [64] and crystal plasticity simulations [65] for nanocrystalline metals, where a major shear band was formed in the samples during deformation. For the CG structure, due to the excellent strain hardening capability of the material, it could deform with a large strain (i.e., $\varepsilon_{u}=44.28\%$) without the presence of apparent strain localization, as shown in Figure 5f, and finally failed as the result of the formation of a neck (Figure 5g). 

**Figure 6:**
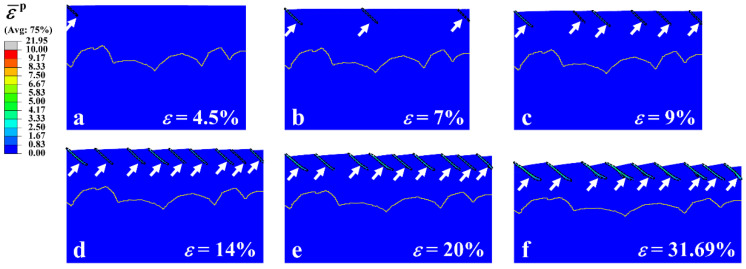
(**a**–**f**) Evolution of shear bands in the gradient Cu with applied strain *ε*. Regions with equivalent plastic strain ${\bar{\varepsilon }}^{p}$ larger than 1 are shown by black dashed lines. Shear bands are indicated by white arrows. The yellow lines are dividing lines between the CG core and the GSL.

For the gradient structure, since the grain size in the surface region of the material was in the nanoscale, shear bands were formed, as indicated by the white arrows in Figure 5d,e. A more detailed illustration of the evolution of shear bands in the gradient Cu is presented in Figure 6, where the applied strain *ε* varies from 4.5% to 31.69%. The dividing lines between the CG core and the GSL are represented by the yellow lines. From Figure 6, it is clear that the shear band in the gradient Cu started to form at a very small, applied strain, e.g., ε=4.5%, which is similar to the NG structure. This result is consistent with the experiments by Yuan et al. [35], in which the shear band at the surface of the gradient IF steel appeared at a very small applied strain of ~1%. However, because of the unique gradient variation of grain size along the thickness direction of the material, the evolution of the shear band in the surface nanocrystalline region was impeded by the coarse grains in the core, and the shear bands were stopped inside the GSL. This phenomenon of coarse grains blocking shear bands has also been observed in heterogeneous nanostructured metals [66]. As *ε* increased, the number of shear bands *N*_s_ gradually increased, e.g., from $N_{s}=1$ at ε=4.5% to $N_{s}=6$ at ε=9%. The number of shear bands in the gradient Cu saturated after ε=14%, where a total number of nine shear bands were formed. This phenomenon is in good agreement with experimental observations [37] where the density of shear bands in the gradient nanostructured Ni showed a trend of first increasing then remaining almost constant during tension. Therefore, the nanograins in the surface of the gradient structure were stabilized by strain delocalization, i.e., shear band multiplication, rather than failing rapidly as in the homogeneous NG structure. Through this mechanism, the gradient structure can achieve a high ductility while taking advantage of the high strength of the NG layers on the surface, resulting in the excellent strength-ductility synergy.

**Figure 7:**
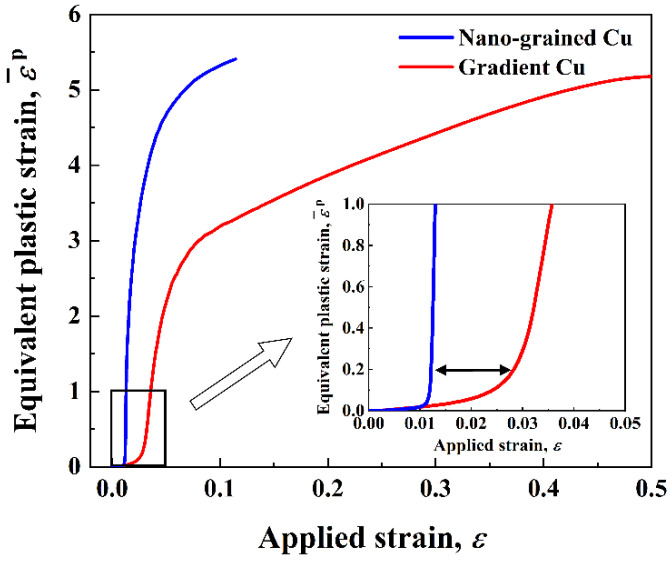
Evolution of the equivalent plastic strain ${\bar{\varepsilon }}^{p}$ at the sites (indicated by the red crosses in Figure 5c,e) inside the shear bands of the NG and the gradient Cu.

In order to further explore the strain delocalization effect in the gradient structure, the plastic strain inside the shear bands of the different structures was analyzed. Figure 7 shows the evolution of the equivalent plastic strain ${\bar{\varepsilon }}^{p}$ at the sites indicated by the red crosses in Figure 5c,e, which are inside the shear bands in the NG and the gradient structures. The results prove that the evolution of shear bands was restrained in the gradient structure. First, compared to the NG structure, the formation of the shear band started later in the gradient structure. That is, as indicated by the black arrow in the inset of Figure 7, the rapid increase of ${\bar{\varepsilon }}^{p}$ in the NG structure started at around ε=0.012, while in the gradient structure the starting point was about ε=0.028. Once ${\bar{\varepsilon }}^{p}$ began to escalate in the NG Cu, it quickly reached a very high value, with just a tiny increase in the applied strain, e.g., from ${\bar{\varepsilon }}^{p}=1$ to ${\bar{\varepsilon }}^{p}=4$ with an increase in *ε* of only 0.02. By contrast, the shear band evolved slowly in the gradient structure. For example, to achieve the same increase in ${\bar{\varepsilon }}^{p}$ as mentioned above, the applied strain *ε* had to be increased by 0.19, which is 9.5 times that of the *ε* increase in the NG structure. This means that the evolution of shear bands was suppressed in the gradient structure. 

### 3.2. Effects of Grain Size Distribution on Shear Band Formation

**Figure 8:**
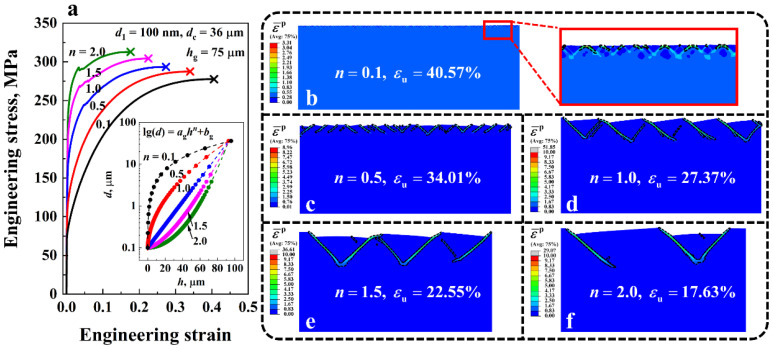
(**a**) Engineering stress-strain curves of the gradient Cu with different grain size distribution index *n* ranging from 0.1 to 2. Variations of the grain size *d* along the thickness direction of the material under different *n* are shown in the inset. (**b**–**f**) Comparison of the shear band patterns of the gradient Cu with different *n* at the uniform elongation $\varepsilon_{u}$. Regions with equivalent plastic strain ${\bar{\varepsilon }}^{p}$ larger than 1 are enclosed by black dashed lines.

Figure 8a shows the engineering stress-strain curves of the gradient Cu with different grain size distribution indexes *n* from 0.1 to 2. Uniform elongation is denoted by the symbol ‘×’. The corresponding deformation behaviors of the gradient structures at uniform elongation are depicted in Figure 8b–f. Regions with equivalent plastic strains ${\bar{\varepsilon }}^{p}$ larger than 1 are enclosed by black dashed lines. Here the GSL thickness *h*_g_, surface grain size *d*_1_, and core grain size *d*_c_ were fixed at 75 μm, 100 nm, and 36 μm, respectively. The grain size distributions along the thickness direction of the material for different *n* are shown in the inset of Figure 8a, in which each dot on the curves represents one homogeneous layer in the GSL of the gradient material. Different from the simulations in the last section, no notch was introduced into the model in the current and subsequent sections since the non-uniform deformation can be induced by the heterogeneous structure in the gradient Cu. The results show that as *n* increases, the yield strength of the gradient material increases, while the ductility decreases. For example, at *n* = 0.1, the yield strength $\sigma_{Y}$ of the gradient Cu was 79.9 MPa, which is nearly one-third of that of the material with *n* = 2, i.e., $\sigma_{Y}=222.06\;\mathrm{MPa}$. However, the ductility of the former ($\varepsilon_{u}=40.58\%$) was more than twice that of the latter ($\varepsilon_{u}=17.63\%$). An important contribution to the high ductility of the gradient Cu with low *n* is the strain delocalization in the surface nanocrystalline region. As shown in the inset of Figure 8b, a large number of shear bands were formed on the surface of the gradient structure when *n* = 0.1. By contrast, only three shear bands were formed at *n* = 2, inducing the low ductility of the material. 

### 3.3. Effect of Thickness of the Gradient Surface Layer on Shear Band Formation

**Figure 9:**
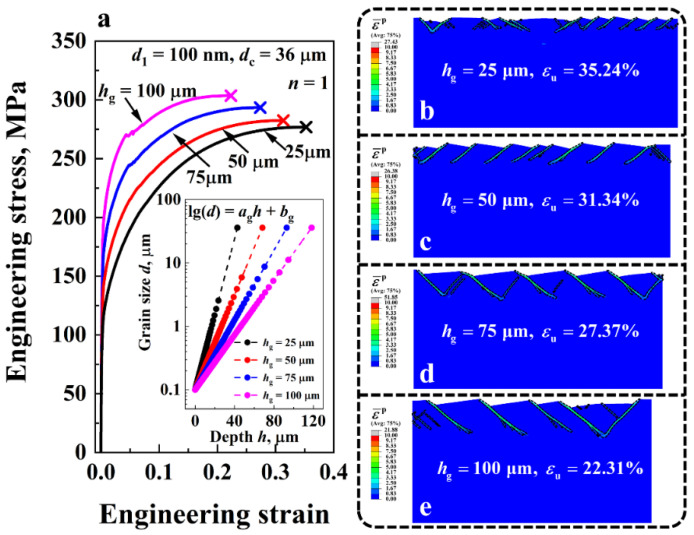
(**a**) Engineering stress-strain curves of the gradient Cu with different GSL thickness *h*_g_ ranging from 43 μm to 118 μm. Variations of the grain size *d* along the thickness direction of the material under different *h*_g_ are shown in the inset. (**b**–**e**) Comparison of the shear band patterns of the gradient Cu with different *h*_g_ at the uniform elongation $\varepsilon_{u}$. Regions where the equivalent plastic strain ${\bar{\varepsilon }}^{p}$ was larger than 1 are enclosed by black dashed lines.

Figure 9a compares the engineering stress-strain curves of the gradient Cu with different GSL thicknesses *h*_g_ ranging from 25 μm to 100 μm. The uniform elongation is represented by the symbol ‘×’. The corresponding deformation behaviors of the gradient structures at the uniform elongation $\varepsilon_{u}$ are shown in Figure 9b–e. Regions with an equivalent plastic strain ${\bar{\varepsilon }}^{p}$ larger than 1 are enclosed by black dashed lines. Here the grain size distribution index *n*, surface grain size *d*_1_, and core grain size *d*_c_ were adopted as 1, 100 nm and 36 μm, respectively. The grain size distributions along the thickness direction of the material for different *h*_g_ are shown in the inset of Figure 9a. The results show that the ductility of the gradient Cu declined as *h*_g_ increased, while its yield strength exhibited the opposite trend of increasing. For instance, from *h*_g_ = 25 μm to *h*_g_ = 100 μm, the uniform elongation $\varepsilon_{u}$ of the material decreased from 35.24% to 22.31%, but the yield strength $\sigma_{Y}$ increased from 106.56 MPa to 187.81 MPa. As can be seen from Figure 9b,c, the difference in the shear band pattern of the gradient Cu with different *h*_g_ is a major cause of the discrepancy in ductility. When *h*_g_ was small, a large number of shear bands were formed at the surface of the material, e.g., *N*_s_ = 24 for the case of *h*_g_ = 25 μm, delocalizing plastic strain in the NG layers. By contrast, only 10 shear bands (Figure 9e) were induced in the gradient structure with a large *h*_g_ of 100 μm, which reduced the ductility of the material.

### 3.4. Effect of Grain Size of the Topmost Layer in GSL on Shear Band Formation

**Figure 10:**
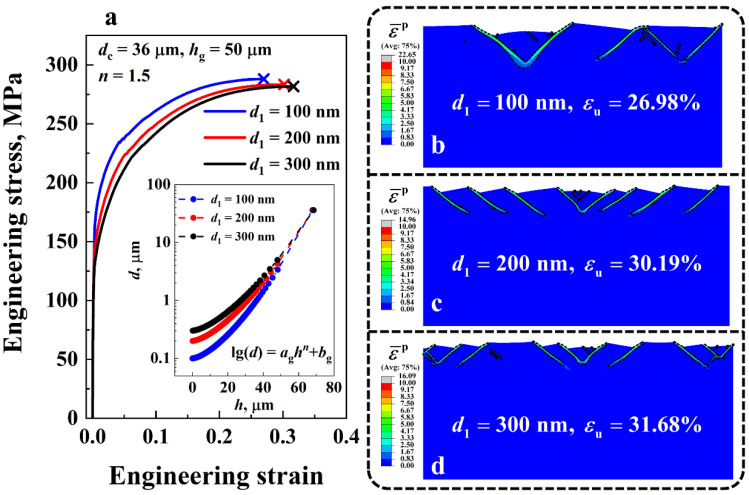
(**a**) Engineering stress-strain curves of the gradient Cu with surface grain size *d*_1_ ranging from 100 nm to 300 nm. Variations of the grain size *d* along the thickness direction of the material under different *d*_1_ are shown in the inset. (**b**–**d**) Comparison of the shear band patterns of the gradient Cu with different *d*_1_ at the uniform elongation $\varepsilon_{u}$. Regions with an equivalent plastic strain ${\bar{\varepsilon }}^{p}$ larger than 1 are enclosed by black dashed lines.

The engineering stress-strain curves of the gradient Cu with three distinct surface grain sizes *d*_1_, i.e., 100 nm, 200 nm and 300 nm, are plotted in Figure 10a. On the curves, the symbol ‘×’ denotes the uniform elongation. The core grain size *d*_c_, the GSL thickness *h*_g_, and the grain size distribution index *n* were set to be 36 μm, 50 μm, and 1.5, respectively. Figure 10b–d presents the corresponding deformation behavior of the gradient Cu with different *d*_1_. It can be seen in Figure 10a that the ductility of the gradient material increased as *d*_1_ increased, while its strength diminished. That is, for the case of *d*_1_ = 100 nm, the uniform elongation $\varepsilon_{u}$ and the yield strength $\sigma_{Y}$ of the material were 26.98% and 155.65 MPa, respectively. When *d*_1_ increased to 300 nm, $\varepsilon_{u}$ and $\sigma_{Y}$ of the material were 31.68% and 128.2 MPa, which are 117% and 82% of those of the values for the case of $d_{1}=100\;\mathrm{nm}$, respectively. The enhanced ductility of the gradient Cu with large *d*_1_ was attributed to the strain delocalization effect at the surface of the material. From Figure 10b–d, we can see that only five shear bands were formed in the case of *d*_1_ = 100 nm. By contrast, there were 13 shear bands in the gradient Cu when *d*_1_ was 300 nm, which is nearly three times the number of shear bands in the gradient Cu with *d*_1_ = 100 nm. The large number of shear bands delocalized the plastic strain in the NG layers of the material, and therefore elevated the ductility. 

**Figure 11:**
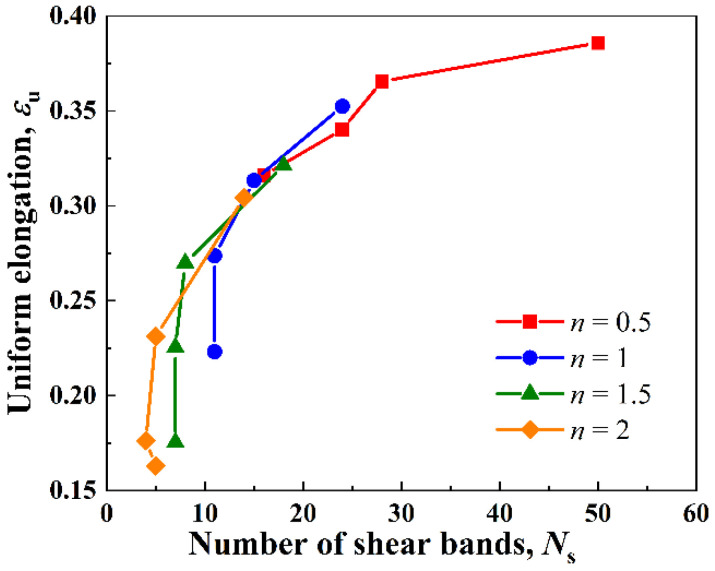
Relation between the number of shear bands *N*_s_ and the uniform elongation $\varepsilon_{u}$ of the gradient Cu.

Figure 11 summarizes the relation between the uniform elongation $\varepsilon_{u}$ and the number of shear bands *N*_s_ of the gradient Cu. Four cases of grain size distribution indexes, i.e., *n* = 0.5, 1, 1.5, and 2, are considered here. For each curve in Figure 11, the GSL thickness *h*_g_ varied from 25 μm to 100 μm. The surface grain size *d*_1_ and the core grain size *d*_c_ were fixed at 100 nm and 36 μm, respectively. The results show that the uniform elongation, i.e., ductility, of the gradient Cu was positively correlated with the number of shear bands formed in the material. The highest ductility i.e., a uniform elongation of ${\bar{\varepsilon }}^{p}=38.58\%$, was achieved in the case with *n* = 0.5 and *h*_g_ = 25 μm, where 50 shear bands were formed in the material.

### 3.5. Verification of the Computational Model

**Figure 12:**
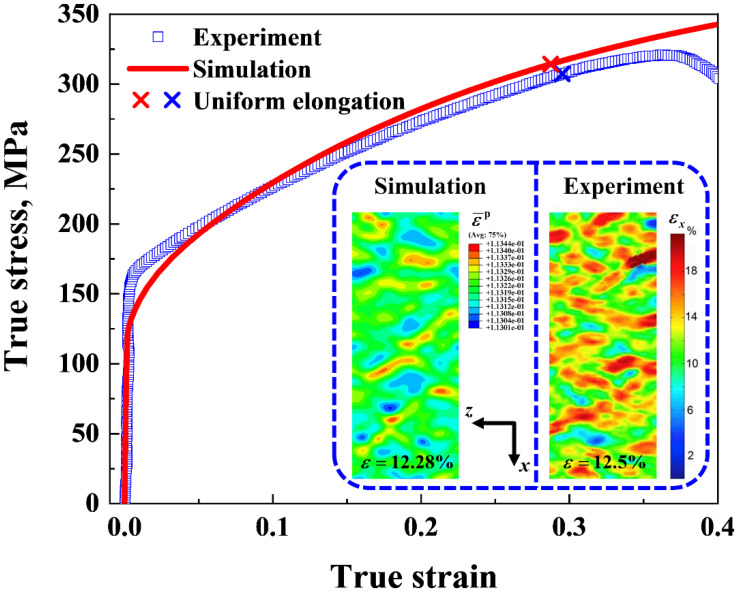
Comparison of the engineering stress-strain curve between the 3D simulation and the experimental data [36]. The strain distribution at the top surface (in the *xz*-plane) of the sample under the applied strain of ~12.5% in both the simulation and the experiment are presented in the inset. The experimental strain map was reprinted from [36] with permission from Elsevier.

Figure 12 shows the comparison of the true stress-strain curves between the 3D simulation and the experimental data [36]. In the simulation, the surface grain size *d*_1_, the core grain size *d*_c_, and the volume fraction of the GSL were adopted from previous experiments [36,67], and were 120 nm, 36 μm and 25%, respectively. The grain size distribution index *n* was set to be 1.13. Figure 12 shows that the simulated stress-strain curve of the gradient Cu was in good agreement with the experimental results. Moreover, the yield strength $\sigma_{Y}$ and the uniform elongation $\varepsilon_{u}$ of the gradient Cu from the simulation were 127.24 MPa and 28.73%, respectively, which are close to those of the experimental data, i.e., 150.48 MPa and 29.51%. Note that the constitutive model used in our simulations was only solid before the uniform elongation was reached, since fracture mechanisms such as the evolution of microcracks [68] were not considered in the model. Therefore, the drop in the experimental stress-strain curve, which appears after the uniform elongation point and is associated with the failure of the material, was not reflected in the model prediction. The inset of Figure 12 shows the shear band pattern at the top surface (in the *xz*-plane; refer to Figure 1 for the coordinate system) of the gradient Cu under the applied strain of ~12.5% from both the simulation and the experiment [36]. It is clear that our model was able to reproduce the shear band morphologies observed in the experiments. 

## 4. Conclusions

In the present study, a computational model was established to study shear band formation in gradient nano-grained Cu with a grain size variation from tens of nanometers at the surface to tens of micrometers at the core. Our simulations revealed that the evolution of a single shear band was suppressed in the gradient material, and strain delocalization through shear band multiplication was achieved in the surface NG region of the material. The gradient material, therefore, possesses a good strength-ductility synergy. We also showed that the number of shear bands in the gradient material was positively related with uniform elongation, and the number of shear bands could be increased by decreasing the grain size distribution index, decreasing the thickness of the grain size gradient region, or increasing the grain size at the material surface. Our model was able to reproduce the stress-strain curve and the shear band patterns of the gradient Cu obtained from experiments. It is worth noting that strain rate may also affect shear band formation in gradient metals, since previous experimental studies have shown that the mechanical behavior of the gradient metals is rate-dependent [34,69]. We will investigate the influence of strain rate on shear banding behavior of gradient metals in a future study.

## Figures and Tables

**Table 1:** Material parameters for the gradient Cu.

Parameter	Symbol	Nanocrystalline	Microcrystalline
Young’s modulus (GPa)	E	121.1	121.1
Shear modulus (GPa)	μ	42.1	42.1
Poisson’s ratio	ν	0.36	0.36
Magnitude of the burgers vector (nm)	b	0.256	0.256
Taylor factor	M	3.06	3.06
Taylor constant	α	0.26	0.37
Hall-Petch slope $(\mathrm{MPa}\cdot m^{1/2}$)	$k_{\mathrm{HP}}$	0.12	0.14
Lattice frictional stress (MPa)	$\sigma_{0}$	48	20
Proportionality factor	ψ	0.0385	0.0166
Dynamic recovery factor	$k_{2}$	17	2.2
Dynamic recovery constant 1	$k_{20}$	12.28	1.6
Dynamic recovery constant 2	$n_{0}$	21.25	21.25
Geometric factor	$k_{3}$	0.45	0.27
Reference grain size (μm)	$d_{e}$	0.82	2.05
Initial dislocation density $(m^{-2}$)	$\rho_{0}$	$7\times 10^{13}$	0
Grain size (μm)	d	<0.5	0.5–36

**Table 2:** Geometrical parameters of the 2D computational model.

Parameter	Symbol	Value
Length of the model (μm)	*L*	200
Total thickness of the model (μm)	*H*	150
Thickness of the GSL (μm)	*h*_g_	25–100
Grain size of the topmost layer (nm)	*d*_1_	100–300
Grain size of the CG core (μm)	*d*_c_	36
Grain size distribution index	*n*	0.1–2

## Data Availability

The data presented in this study are available on request from the corresponding author.
